# Does sediment composition sort kinorhynch communities? An ecomorphological approach through geometric morphometrics

**DOI:** 10.1038/s41598-020-59511-4

**Published:** 2020-02-13

**Authors:** Diego Cepeda, Dolores Trigo, Fernando Pardos, Nuria Sánchez

**Affiliations:** 1 Universidad Complutense, Department of Biodiversity, Ecology and Evolution, Madrid, 28040 Spain; 2 Institut Français de Recherche pour l’Exploitation de la Mer, Deep-sea Laboratory, Plouzané, 29280 France

**Keywords:** Zoology, Ecology, Evolutionary ecology, Evolution

## Abstract

Ecomorphology studies the relationship between organisms’ morphology and environment features. To better understand whether the shape of the body and the appendages involved in the movement is correlated to sediment composition in meiofaunal organisms, we study the evolved morphological adaptations to environment in selected taxa of the phylum Kinorhyncha: the allomalorhagid families Dracoderidae and Pycnophyidae, and the cyclorhagid genus Echinoderes. The selected taxa include the most diverse groups of Kinorhyncha worldwide, representing the 75.5% of the total phylum diversity. Widened, plump bodies and lateral terminal spines may be adaptive for species living in coarse, more heterogeneous sediments, as they could maintain a more powerful musculature to actively displace the sediment grains applying a greater force. Conversely, slender, vermiform bodies and lateral terminal spines would represent an adaptation of species inhabiting fine, more homogeneous sediments where there would not be much need to exert a high force to displace the sediment particles, and a more vermiform shape would even favour the burrowing of the animal through the smaller interstices. The studied kinorhynch taxa would also be adapted to the higher velocity of the sea-water and the intense erosion and transportation of heterogeneous sediments by possessing more robust bodies, avoiding getting laid off substratum under these conditions. These findings provide evolutionary evidence that body shape in the studied kinorhynch groups is adapted to environment.

## Introduction

Morphological adaptations are frequently a response of ecological pressures and changes in environmental variables^1,2^. Ecomorphology can be defined as the study of the relationship between organisms’ morphology and ecological features^3,4^. Indeed, environmental heterogeneity is one of the main promoters of morphological variation in animals that inhabit changeable habitats^5,6^. In the context of marine ecosystems, meiobenthic habitats possess complex, dynamic interactions that are intricately combined and influenced by numerous abiotic factors^7^. The structure of the sediment is one of the main meiobenthic abiotic parameters, performing a leading role in meiobenthic ecology since its features influence the degree of accessibility of meiofaunal organisms^8,9^.

Soft sediments are composed of inorganic particles, organic matter and pore water, so meiobenthic organisms are strongly affected by their variations^10^. As for instance, the grain size of the sediment inhabited by the organisms determines the relative availability of interstitial spaces and subsequently influences the abundance and composition of the meiofaunal communities^11^. Morphological and size adaptations of meiofauna to grain size have been evidenced in different groups. Meiofauna can be divided in several categories regarding the different way of movement through the sediment particles: interstitial forms, burrowers, epibenthic organisms and hyperbenthic taxa. Interstitial meiofauna moves among sedimentary grains without displacing them, whereas burrowing meiofauna actively displace the particles, usually with body structures acting as spades and moved by muscles^10^. Most interstitial taxa (*e.g*. tardigrades, some harpacticoid copepods and nematodes, a few ostracods such as Xestoleberididae, and most gastrotrichs and annelids) are stouter and plumper in finer sediments where they need to dig through the sediment particles or live as sediment dwellers near the surface, whereas slender, vermiform species tend to inhabit in coarser sediments where they can move more easily through the interstitial space^12–15^. However, some exceptions to this can be found, as certain interstitial taxa (*e.g*. the gastrotrich genus *Musellifer* Hummon, 1969, or even some kinorhynchs such as the genera *Cateria* Gerlach, 1956 and *Franciscideres* Dal Zotto *et al*., 2013, or some species of *Cephalorhyncha* Adrianov in Adrianov and Malakhov, 1999) live in fine sediments and are rather slender and vermiform. Burrowing meiofauna (*e.g*. loriciferans, most kinorhynchs and ostracods, and some annelids, harpacticoid copepods and nematodes), that moves by active displacement of the sediment, is more frequent in finer sediments, and relationships between grain size and body shape are much more uncharted^16–18^. Other categories of meiofaunal organisms must also be mentioned, such as the epibenthic forms (*e.g*. gastropods, some foraminiferans such as the *Symbiodinium* Freudenthal, 1962 group), which live on top of other meiofaunal organisms or algae, or the hyperbenthic taxa (most copepods, nauplii, some annelids, nematodes and bivalves) that are able to swim through the water column, having both a more reduced relationship with sediment features^7,14^.

On the other hand, the influence of organic matter on meiofaunal communities has been widely studied from a trophic point of view as it is fundamental for the productivity of meiobenthic communities^19^, but its possible role in the organism shapes still remains unexplored. The carbon-to-nitrogen ratio (C/N) is a good measure of the quality of detritus whose accumulation over sediment usually changes the physical properties of the latter, acting as a cementing agent^20^. Moreover, the C/N ratio also gives information on the state of the decomposition processes, as it depends on several factors including sedimentary features, rate of microbial degradation, column water productivity and terrestrial inputs^21,22^. Finally, the hydrogen ion concentration (pH) is also of relevance in meiobenthic ecology as it can induce morphological deformations and act as limiting factor for many organisms^8,23^.

In this regard, kinorhynchs are an ideal model to study morphology-sediment relationships since this phylum is mainly composed of burrowing meiofaunal species that inhabit a wide variety of oceanic soft sediments^24,25^. The main aim of the present paper is to determine whether and how sediment features (grain size, content of organic matter and pH) affect body shape and size of kinorhynchs, through a geometric morphometrics approach using selected kinorhynch taxa: the allomalorhagid families Dracoderidae (2.3% of the total phylum diversity) and Pycnophyidae (31% of the total phylum diversity), and the cyclorhagid genus *Echinoderes* Claparède, 1863 (42.2% of the total phylum diversity). Likewise, we test if shape and size of two kinds of cuticular appendages are also affected by sediment: lateral terminal spines (LTS) and primary spinoscalids. Primary spinoscalids are used by kinorhynchs to actively move through the sediment^24,25^, whereas the LTS, of still unclear function, are in constant contact with the sediment, being very conspicuous and large in most of the groups and thus forced to move through the sediment particles.

## Results

### Sediments composition

Most of the analysed sediment samples (*n* = 16) consisted in sand, with an average size of 49.44–327.8 μm. Seven samples were defined as mud, with an average size of 9.5–27.52 μm, and only two were dominated by gravel, with an average size of 56.96–1353.9 μm. Values of sorting (*σ*) were generally low (mean of *σ* = 4.085), and hence most of the sediment samples were poorly sorted, with the sediment spread over a large range of size classes (*i.e*. more heterogeneity). However, some samples had high values of sorting and were well sorted (*σ* up to 16.54), with most of the sediment confined to a few size classes. Values of skewness (*Sk*) indicated a general trend to the asymmetry in the spread of the particle sizes towards low diameters (mean of *Sk* = −0.243), meaning that the samples contain more categories of particles with sizes under the average value (*i.e*. more categories of finer particles). Nevertheless, some samples had positive values of *Sk* with more categories of coarser particles above the average size. Finally, values of kurtosis (*K*) revealed that most of the samples are leptokurtic, with a low concentration of the particles relative to the average size (mean of *K* = 1.704). Some samples were determined as platykurtic (*K* < 1), with particles very concentrated relative to the average size, as well as mesokurtic (*K* ≈ 1). In summary, though there is a majority of sandy, quite heterogeneous, leptokurtic sediments, the set of samples are of relative variability.

Regarding the content in organic matter, range of C/N ratio was wide, from 7.71 to 61.26. Most of the analysed samples were poor or very poor in organic carbon content (%_*C*_ < 2.0), except the samples 4 and 5 from Algeciras Bay (%_*C*_ = 2.2821 and 2.2356, respectively; see Supplementary Information). Regarding the content in nitrogen, the samples were also poor or very poor (%_*N*_ < 0.15), except the samples 4 and 5 from Algeciras Bay again *(*%_*N*_ = 0.1821 and 0.1649, respectively; see Supplementary Information). The generally low C/N ratio values seem to be consequence of the poor content in nitrogen of most of the analysed samples. Moreover, an important input of terrestrial organic matter was detected in most of the sampled localities, with high values of C/N ratio (>10)^26^. A single exception was found at one locality of Jamaica (locality 2) where organic matter of marine origin seemed to be dominant, showing a low value of C/N ratio (between 4 and 10) (see Supplementary Information). pH varied from 6.677 to 8.883, with most of sediment samples showing alkaline values.

### Body shape

**Figure 1:**
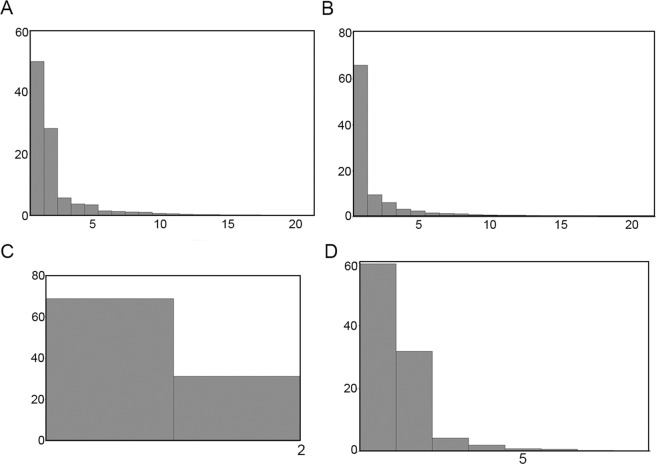
Principal component axes that explain the variance in the symmetrical component of the body shape (**A**), the asymmetrical component of the body shape (**B**), the LTS shape (**C**) and the primary spinoscalids shape (**D**). X axis represents number of principal components, Y axis represents % explained by principal components.

**Figure 2:**
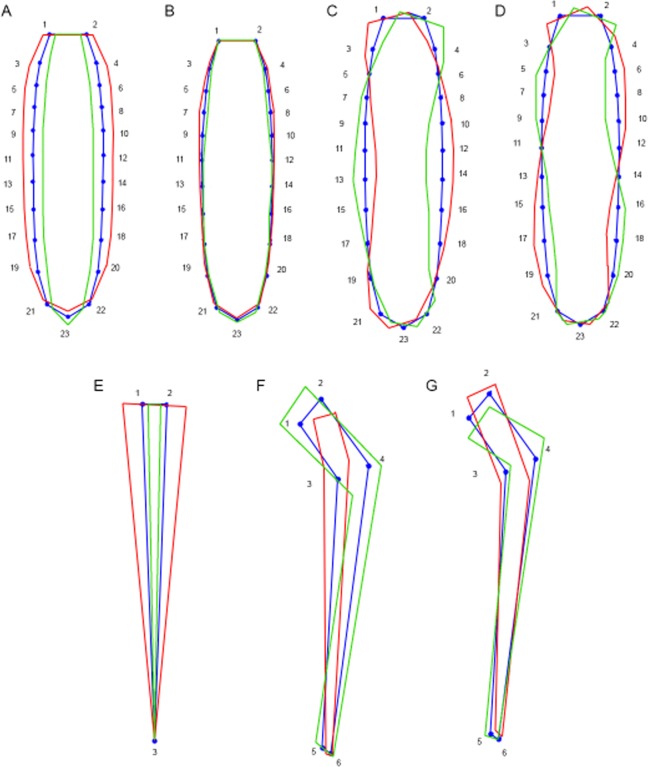
Wireframe representations of the variation in shape of sternal plates (**A–D**), LTS (**E**) and primary spinoscalids (**F,G**) along the principal components that explain most of the variance. Blue wireframes represent the mean shape observed across all sample individuals, and red and green wireframes represent the most extreme shapes. (**A**) PC1 of symmetrical component of sternal plates; (**B**) PC2 of symmetrical component of sternal plates; (**C**) PC1 of asymmetrical component of sternal plates; (**D**) PC2 of asymmetrical component of sternal plates; (**E**) PC1 of LTS; (**F**) PC1 of primary spinoscalids; (**G**) PC2 of primary spinoscalids.

The two first axes of the principal component analysis (PCA) explained a total of 78.374% of the symmetric (50.044% by PC_1_ and 28.330% by PC_2_) and 75.504% of the asymmetric (65.833% by PC_1_ and 9.671% by PC_2_) components of the variation in shape across specimens (Fig. 1A,B). Regarding the symmetric component, specimens with positive PC_1_ and PC_2_ values tended to have a body shape that was more widened and plumper, whereas those specimens with negative PC_1_ and PC_2_ values had a tendency towards a slender, more narrowed (vermiform) body shape, as showed by the wireframe graphs (Fig. 2A,B). Here, we will refer to these two body shape extremes as “stouter and plumper” and “vermiform and slender”, respectively.

Linear mixed models (LMMs) and generalized linear mixed models (GLMMs) yielded that the variance in body shape by the symmetric component was 27.66% explained by PC_1_ and 21.95% by PC_2_, as determined by the coefficients of determination (Table 1). Average size, skewness and kurtosis were the only sediment variables that contributed to explain the shape variance of the model (Table 1). According with the model, stouter and plumper specimens are more likely found in sediments with a coarse average size of particles with more representation of coarser categories (as indicated by the positive values of skewness) and a relatively high value of heterogeneity in particle sizes (as indicated by the positive values of kurtosis). On the opposite, vermiform and slender specimens are more abundant in sediments with a fine average size of particles with more representation of finer categories (as indicated by the negative values of skewness) and a relatively low value of heterogeneity in particle sizes (as indicated by the negative values of kurtosis).

**Table 1:** Coefficients of linear mixed models and generalized linear mixed models of body and LTS shape analyses. Only variables with significant results (*p* < 0.05) are included. Abbreviations: C/N, carbon-nitrogen ratio; *K*, kurtosis; *R*^2^, coefficient of determination; *Sk*, skewness; StdE, standard error; *t*, t-value; *X*, average size; %_*EXP*_, % explained by random-effect component.

Model	Variable	Estimate	StdE	*t*	*R*^2^	%_*EXP*_
PC_1Sym_	Intercept	1.66e-01	1.09e-01	1.52	0.2766	58.96
	*X*	1.01e-04	5.46e-05	1.85		
	*Sk*	3.46e-02	1.95e-02	1.78		
	*K*	1.31e-02	4.68e-03	2.79		
PC_2Sym_	Intercept	−7.289e-02	7.33e-02	−0.994	0.2195	27.55
	*X*	5.986e-05	1.807e-05	3.312		
	*Sk*	6.747e-02	1.132e-02	5.962		
	*K*	7.652e-03	3.315e-03	2.308		
PC_1Asym_	Intercept	−1.037e-01	5.744e-02	−1.805	0.4685	0
	pH	1.327e-02	7.161e-03	1.853		
PC_2Asym_	Intercept	6.739e-03	2.188e-02	0.308	0.5218	0
PC_1lts_	Intercept	7.17e-02	5.76e-02	0.213	0.2066	32.12
	*Sk*	1.52e-02	8.97e-03	0.090		
	C/N	−5.03e-04	2.06e-04	0.015		

**Figure 3:**
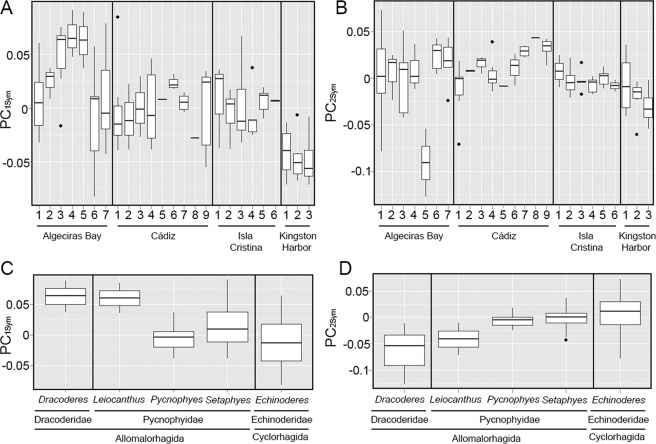
Box-and-whisker plots of variation in symmetric body shape between localities (**A,B**) and random-effect (*i.e*. phylogenetic influence) component (**C,D**). The boxplots show the median (middle line), quartiles (boxes), 1.5 times the interquartile range (whiskers) and extreme values (dots).

Additionally, vermiform and slender kinorhynchs were mainly found in the samples from Jamaica, whereas samples from the Iberian Peninsula resulted in a wide range of shapes but usually more robust than the former ones (Fig. 3A,B). However, this result has to be taken with caution, since the Jamaican specimens are represented only by three localities around the same bay.

Phylogeny resulted of high influence, with 58.96% for PC_1_ and 27.55% for PC_2_ of the variance not explained by the fixed-effect component of the models (Table 1). These results showed an important genetic effect in body shape, as also showed by the boxplots (Fig. 3C,D). Thus, the cyclorhagid genus *Echinoderes* was characterized by a slender, more vermiform shape, with a mean value of PC_1_ lower than the allomalorhagid families Dracoderidae and Pycnophyidae (Fig. 3C). The allomalorhagid genera *Dracoderes* Higgins & Shirayama, 1990 and *Leiocanthus* Sánchez *et al*., 2016 showed the most robust and plumpest body shape (Fig. 3C).

Concerning the asymmetrical component, specimens with positive or negative PC_1_ and PC_2_ values tended to suffer different kinds of deviations (slight twists and zigzags on sternal plates) from the bilateral symmetry (Fig. 2C,D). Only the sediment pH seemed to influence these deviations of the symmetrical pattern, affecting the PC_1_ and explaining 46.85% of the total variance (Table 1). We found a positive correlation between pH and PC_1_, with more alkaline values causing deviations from bilateral symmetry. Phylogeny did not show any influence on the asymmetrical component of body shape, as the random-effect component of the model was not found as an explicative factor.

### LTS shape

Variation in shape of LTS is mainly explained by the first PCA axis (68.715%) (Fig. 1C). Positive values of PC_1_ defined widened and stout spines, while negative values tended to define slender and narrowed spines (Fig. 2E).

**Figure 4:**
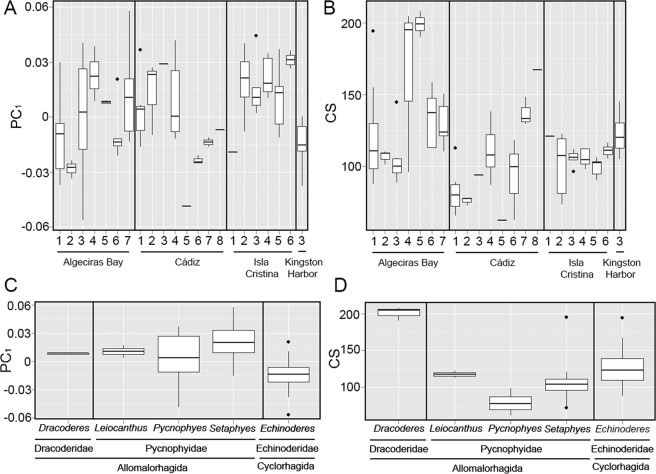
Box-and-whisker plots of variation in LTS shape between localities (**A**) and random-effect (*i.e*. phylogenetic influence) component (**C**), and variation in LTS size between localities (**B**) and random-effect (*i.e*. phylogenetic influence) component (**D**). The boxplots show the median (middle line), quartiles (boxes), 1.5 times the interquartile range (whiskers) and extreme values (dots).

The only sediment variables that influenced in modelling LTS shape were skewness, kurtosis and C/N ratio, explaining a 20.66% of the total variance of PC_1_ (Table 1). Stouter and more widened spines tended to be correlated with sediments with more heterogeneity of particle sizes, with special abundance of coarser categories, and with lower C/N ratios (more content in organic nitrogen), and vice versa. Regarding the localities, we found a huge variety of LTS shapes with no apparent differences between the Iberian Peninsula and Jamaica (Fig. 4A). The random-effect component only had a high influence in LTS shape regarding the PC_1_ (*%*_*EXP*_ = 32.12). Indeed, the cyclorhagid genus *Echinoderes* showed the trend of more elongated, slender spines than the allomalorhagid families Dracoderidae and Pycnophyidae, whose mean values determined the presence of stouter, more widened structures (Fig. 4C).

### Primary spinoscalids shape

Variation in shape of primary spinoscalids was explained in a 91.605% by the first two PCA axes (PC_1_ = 59.68% and PC_2_ = 31.925%) (Fig. 1D). Positive values of PC_1_ defined scalids with a proportionally shortened and narrow basal sheath and a more acicular tip, whereas negative values were related to scalids with a proportionally elongated and wide basal sheath with a distally rounded tip (Fig. 2F). Variation of PC_2_ defined scalids with more elongated but still wider basal sheaths and a more acicular tip with positive values, and proportionally shortened but narrower basal sheaths and a rounded distal tip with negative values (Fig. 2G).

None of the sediment variables showed a significant influence on the shape variation.

### Size

Centroid size (CS) of sternal plates varied between 147.99 and 1136.57 (653.08 ± 301.12), *i.e*. the largest kinorhynchs were almost eight times larger than the smallest ones. The smallest kinorhynch belonged to the species *Leiocanthus lageria* (Sánchez *et al*., 2013), and the largest one to the species *Setaphyes dentatus* (Reinhard, 1881). There was no significant influence of sediment variables on body CS.

CS of LTS varied between 62.11 and 207.94 (113.73 ± 29.49), meaning that the biggest LTS were more than three times larger than the smallest ones. *Pycnophyes communis* Zelinka, 1908 was the species with the smallest LTS, while *Dracoderes gallaicus* Sørensen *et al*., 2012 possessed the largest ones (Fig. 4D). We found influence of sorting, skewness and kurtosis on LTS CS, as the fixed-effect component of the model explained together a 23.53% of the total observed variance (Table 2). Additionally, phylogeny resulted of high influence as the random-effect component of the model explained 65.44% of the variance. We did not found any particular difference between samples from the Iberian Peninsula and the sample from Jamaica (Fig. 4B). In summary, we found specimens with larger LTS in sediments with high heterogeneity of particle sizes and more content of finer sediments.

**Table 2:** Coefficients of generalized linear mixed models of LTS size analyses. Only variables with significant results (*p* < 0.05) are included. Abbreviations: *K*, kurtosis; *R*^2^, coefficient of determination; *Sk*, skewness; StdE, standard error; *t*, t-value; %_*EXP*_, % explained by random-effect component.

Variable	Estimate	StdE	*t*	*R*^2^	%_*EXP*_
Intercept	−0.0039	0.0051	−0.7605	0.2353	65.44
*σ*	0.00008	0.0001	0.7833		
*Sk*	−0.0022	0.0008	−2.9397		
*K*	0.0004	0.0002	1.8871		

CS of primary spinoscalids varied between 27.17 and 76.25 (43.35 ± 11.87), meaning that the largest primary spinoscalids were almost three times larger than the smallest ones. *Echinoderes hispanicus* Pardos *et al*., 1998 was the species with the smallest primary spinoscalids, whereas *Dracoderes gallaicus* had the largest ones. There was no significant influence of sediment variables on primary spinoscalids CS.

## Discussion

Meiofaunal organisms are tremendously dependent of sediment and therefore reveal the effect of sediment structure and composition on morphology as crucial to better understand animals-habitat interactions. For our group of study, the allomalorhagid families Dracoderidae and Pycnophyidae and the cyclorhagid genus *Echinoderes* (phylum Kinorhyncha), we found relationships between body and LTS shape with some of the analysed sediment variables. Additionally, the phylogeny contributed to the variation in shape of kinorhynchs as well.

Body shape adaptations of meiofauna to grain size have been mainly explored in interstitial taxa. Sediments with coarser size of particles usually host slender, more vermiform species as they have to move through tight interstitial spaces, whereas finer sediments are inhabited by stouter species whose body enables them to penetrate more easily through the particles, acquiring a movement similar to that of burrowers^12–15^. However, relationships between body shape and sediment composition still remains unexplored for burrowing meiofauna. Kinorhynchs, as meiofaunal burrowers mainly, use the introvert scalids to move through the sediment^17,25,27^.

We found statistically significant influence of average size, skewness and kurtosis of sedimentary particles over body shape of the analysed kinorhynch taxa. The analyses showed that kinorhynch species inhabiting coarser sediments with a high variety of different particles’ sizes tended to be stouter and plumper compared to those inhabiting finer, more homogeneous sediments, whose bodies were slender and more vermiform. These results seem to go, at first sight, against those above mentioned for the interstitial meiofauna. However, the analysed kinorhynch taxa are representatives of the burrowing meiofauna that move actively through the sediment displacing the grains with the introvert scalids^24,25^. In this context, a more robust and plumper body may suppose an adaptive advantage for the species that live in coarser sediments, allowing to maintain a more powerful musculature to displace the sediment particles by generating a greater force. Indeed, for the interstitial taxa that act to some extent as burrowers in finer sediments, plump and robust bodies suppose an adaptation for this active movement through the sediment, as previously explained. In parallel, the possession of a slender, vermiform body in species inhabiting finer sediments would also be adaptive, since it would not be necessary to apply much force to move the finer sediment particles, and this body morphology would even facilitate the burrowing through the smallest interstices by simple movements of the body combined with that of the introvert scalids. Indeed, fine-grained sediments of moderate to high water content show the phenomenon of thixotropy, where a small force against the sediment is enough to allow sediment displacement^10^. It remains to be seen if other kinorhynch taxa not included in the present study (*e.g*. Franciscideridae, Kentrorhagata, Xenosomata) agree with the results herein obtained for Dracoderidae, Pycnophyidae and *Echinoderes*, or if they contrarily exhibit different patterns of body morphological adaptations to sediment.

Heterogeneity of grain sizes, very different from those of the average (kurtosis), also seems to favour the presence of more robust, plump species. These sediments correspond to gravel and gravelly sand, reflecting the presence of many different categories of coarser sizes. This result seems to also support the previously proposed hypothesis. Heterogeneity of inorganic particles dominated by coarse sediments also reflects a heterogeneity of sedimentary processes, with variable and strong depositional currents^10,27–29^. In these areas of high current velocity, with intense erosion and transportation, meiofaunal organisms must be capable of rapid reburial, favouring the presence of stout and plump burrowing species in coarser sediments.

Regarding the phylogenetic component of the models, body shape differences among classes and families are strongly influenced by genetics. In this context, some kinorhynch taxa with slender and more vermiform bodies, such as the allomalorhagid genera *Franciscideres* Dal Zotto *et al*., 2013 and *Gracilideres* Yamasaki, 2019, or the cyclorhagid genera *Cateria* Gerlach, 1956, *Triodontoderes* Sørensen and Rho, 2009 and *Zelinkaderes* Higgins, 1990 have been found inhabiting relatively coarse sediments, except *Triodontoderes lagahoo* Cepeda *et al*., 2019 and *Zelinkaderes floridensis* Higgins, 1990. This could be due to the existence of different adaptation patterns in the remaining kinorhynch taxa not included in the present study. Although the allomalorhagid families Dracoderidae and Pycnophyidae plus the cyclorhagid genus *Echinoderes* constitute an extensive part of the total phylum diversity (75.5%), still another great part of kinorhynch morphological diversity is not included herein., which could possess different morphological adaptations to sediment.

It has been also hypothesized that slender, vermiform kinorhynchs tend to inhabit coarser sediments as an adaptation to the interstitial environment^30^. However, this hypothesis is mostly based in the presence of a thin and flexible cuticle that would make the animal more bendable when moving through the sediment particles, rather than in the shape of the body. Thus, it is likely that a combination of morphological features, including body shape and flexibility of the cuticle, among others, are responsible from defining the adaptive process of kinorhynchs to the different types of substrata.

Fluctuating asymmetry (*i.e*. departures from perfect bilateral symmetry) usually occurs due to the incapacity of the organisms to contain disorders from environment or endogenous conditions during its development, leading to a lesser reproductive success and survival rate^31^. According to our results, body asymmetry of analysed kinorhynchs is affected by pH, reflecting the largest deviations from the bilateral pattern of the sternal plates under values of pH below 7.0 (acidic) and above 8.5 (strongly alkaline) as well (see Supplementary Information). In marine sediments, pH between 7.5–8.5 is well-buffered against pH oscillations, but lower or higher values, combined with other stress factors (*e.g*. high temperatures, extreme salinity, etcetera) may be detrimental for meiofauna^7^. Indeed, previous studies have shown that meiofauna experiences episodes of high mortality after exposure to recurrent pH changes^32–35^.

On the other hand, animals with exoskeletons containing chitin, such as crustaceans and molluscs, suffer a significant loss of chitin under acidic pH values^36,37^. Kinorhynchs also possess an external cuticle with a chitinous basal layer^25^, and deformations in body morphology could be induced under acidic conditions. As for the possible effect of alkaline pH, it is likely that too alkaline (>8.5) values are not able by themselves to cause deviations from the bilateral symmetry of kinorhynchs. However, combined with other environmental factors such as episodes of increasing temperature and salinity, alkaline pH could induce deformations of kinorhynch sternal plates, leading to asymmetrical patterns.

The lack of genetic basis of the observed asymmetry in kinorhynchs is in accordance with the aforementioned idea about deviations from the bilateral symmetry, as these deviations usually lead to low rates of fitness and biological success in animals.

LTS are elongate, basally articulated, distally pointed cuticular appendages present in lateroventral position on segment 11 of most kinorhynch species^25^. The function of these spines still remains unknown, but they are the most conspicuous cuticular structures as they tend to be the largest ones compared to other appendages, projecting well beyond the end of the trunk. Another conspicuous cuticular appendage is the midterminal spine, present in some kinorhynch taxa such as the cyclorhagid orders Kentrorhagata and Xenosomata, or the allomalorhagid families Franciscideridae and Neocentrophyidae. In any case, LTS are forced to move through the sediment interstices accompanying the general movement of the animal.

According to our results, species with more shortened and widened LTS seem to occur in sediments with a wide range of different coarse particles (*i.e*. dominated by coarse sediments), whilst species with slender and more narrowed LTS tend to inhabit in substrata with a wide range of different fine particles (*i.e*. dominated by fine sediments). These results are similar to those obtained for kinorhynchs’ body shape. More robust and widened LTS could allow kinorhynchs of the analysed taxa better moving through the sediment particles in coarser sediments, actively moving and displacing the grains by exerting a greater force. In fact, LTS are the only cuticular appendages of Kinorhyncha (together with the midterminal spines) that are linked to internal muscles, meaning that the animals are able to move them^38–40^. Additionally, coarser sediments are usually a result of strong currents, so the presence of more robust, widened LTS could allow kinorhynchs clinging more tightly to the sediment particles under episodes of high hydrodynamics. It is important to note that the obtained results for the LTS adaptive shape are not necessarily applicable to kinorhynch taxa bearing an also conspicuous midterminal spine, which have not been included in the present study. Most of these kinorhynchs with a midterminal spine, also linked to internal muscles, inhabit fine sediments (Neuhaus, 2013), and a possible morphological adaptation to sediment of this cuticular appendage remains to be explored.

Moreover, species with shortened and widened LTS were mostly found in sediments with more content in organic nitrogen and more likely of marine origin, whereas species with slender and narrowed LTS were mainly found in sediments with more content in organic carbon and a significant input of terrestrial organic matter. The different proportions of carbon and nitrogen influence the abundance and composition of the micro and meiobenthos communities, influencing in the possible food sources for kinorhynchs and the complex biological interactions between the different taxa, and this could lead to the observed differences in LTS shape, rather than carbon and nitrogen affecting the physical properties of the sediment^8^.

Finally, a relationship between LTS’ size and three sediment variables (sorting, skewness and kurtosis) was found. Thus, larger LTS are related to sediments with many different size categories (heterogeneous) and a certain dominance of finer categories, while species with smaller LTS inhabit more homogeneous sediments with a certain dominance of coarser categories. Heterogeneous grain size distributions in marine sediments is usually linked to dynamic, intensely eroded areas, as mentioned above, or influenced by processes of bioturbation^41^. Additionally, heterogeneous sediments possess a more efficient grain packing than homogeneous sediments of similar size^42^. Sediment packing influences the amount of water that can be stored in sediment and its ease of circulation, the degree to which dissolved materials can be hosted and the strength of the sediment under shearing load^43^. In this context, the presence of larger LTS in such heterogeneous sediments, with more dynamism of sea-water circulation and bioturbation processes together with a larger amount of organic matter and other dissolved materials that may hinder movements, could facilitate the displacement of the animal through the interstices and furthermore favour its anchoring to the sediment particles if needed.

None of the sediment variables showed a significant influence on the primary spinoscalids shape. This result is striking taking into account that scalids are the main kinorhynch appendages used for displacement^25^ and consequently they should be influenced by sediment structure and composition. However, this could be explained by the lower sample size of primary spinoscalids’ micrographs (*n* = 22) compared to that of body shape (*n* = 127) and LTS (*n* = 99).

## Methods

### Sampling and dataset

Samplings were done using a meiobenthic dredge in two different campaigns: one around the Iberian Peninsula, from February to November 2011 (22 localities), and one off Jamaica, in March 1976 (three localities). Samples from the Iberian Peninsula were collected by Dr J. Benito, Dr M. Herranz, Dr F. Pardos and Dr N. Sánchez in three main regions: Algeciras Bay (western Mediterranean Sea), Cádiz and Isla Cristina, Huelva (north-eastern Atlantic Ocean), whereas samples from Jamaica were collected by Dr R. P. Higgins in Kingston Harbor (Caribbean Sea). For a complete list of species, number of specimens per species and data on sampling localities see Supplementary Information. Dredges are really useful for collecting large samples of meiofaunal organisms rather than more quantitative methods such as corers that usually collect too low numbers of specimens, but they may present some problems when applying granulometric methods in the collected sediment. Meiobenthic dredges cannot be always related to sediment area, because they usually mix the different patches of sediment that are in a certain area, but we tried to minimize this bias by getting the dredge in circles around the same area of sediment and selecting the sampled areas following nautical charts of bottom sea sediments to only sample in those locations with a rather homogeneous sediment (not split into patches).

Each sediment sample was firstly used to extract meiofaunal organisms by the bubble-and-blot method, then fixed and preserved in a neutralised formalin solution to prevent the organic matter from decaying, for granulometric, organic matter and pH analyses. A total of 127 kinorhynch specimens of the two extant classes (Allomalorhagida, *n* = 62; Cyclorhagida, *n* = 65) were obtained and studied, including representatives of three families (allomalorhagid Dracoderidae, *n* = 3, and Pycnophyidae, *n* = 59; cyclorhagid Echinoderidae, *n* = 65) accommodated into five genera (*Dracoderes*, *n* = 3; *Echinoderes*, *n* = 65; *Leiocanthus*, *n* = 2; *Pycnophyes*, *n* = 16; *Setaphyes*, *n* = 41). It must be noticed the lack of part of the diversity of the phylum Kinorhyncha in the studied samples, including the allomalorhagid families Franciscideridae and Neocentrophyidae, and the cyclorhagid orders Kentrorhagata and Xenosomata. Indeed, the results herein obtained are only applicable to the allomalorhagid families Dracoderidae and Pycnophyidae, and the cyclorhagid order Echinorhagata, which are, on the other hand, the species-richest taxa within Kinorhyncha.

### Photography

Light micrographs of kinorhynch specimens were obtained using an Olympus DP-70 camera attached to an Olympus BX51-P microscope with differential interference contrast (DIC) optics. We followed the subsequent cautions to avoid different kind of biases. Firstly, only adult males were photographed to eliminate morphological variance due to sex and/or developmental stage. We selected males instead of females to have a higher number of sediment samples.

Regarding photographic distortions, we minimized measurement shape errors due to 2D photographs limitations by selecting only those specimens that were as flattened as possible^44^. 2D photographs obscure the variance of a dataset as they eliminate the Z dimension of depth variability^45–48^. The referred flattening does not influence the structures whose morphological variation we wanted to analyse (*i.e*. sternal plates, LTS and primary spinoscalids), since they are in themselves quite flattened (for further information see below *Geometric morphometrics* subsection). Therefore, those specimens that had not been sufficiently flattened during the mounting process for light microscopy (LM) were discarded. Furthermore, barrel and pincushion distortions may appear when taking a photograph, causing the centre of straight lines to bow out toward or bend inward the edges of the image respectively^49^. We avoided the use of different cameras and lenses as well as changing the camera settings and the placement of the specimens near the margins of the photograph to evade the aforementioned distortions.

### Sediment structure and composition

Sediment samples, originally preserved in a neutralized formalin solution, were air-dried to remove remains of formalin. Sediment granulometry and pH in H_2_O were determined following the methods of Guitián & Carballas^50^. Particle size scales applied in the present study follow those adopted by Blott & Pye^51^. According to this criteria, gravel is defined as particles >2 mm diameter, sand as particles from 2 mm to 63 μm, and silt (*i.e*. mud) as particles from 63 μm to 4 μm^51^. Particles defined as clay (<4 μm) were not found in any of the analysed samples, so the term mud used hereinafter refers to silt particles. In each of these main categories of sediment, a series of subclasses were defined to cover the different grain size intervals^51^, which are frequently used in marine sediment works. Four parameters were used to describe grain size distribution: average size, sorting, skewness and kurtosis. Sorting is the spread of the sizes around the average, skewness describes the preferential spread to one side of the average and kurtosis analyses the degree of concentration of the grains relative to the average. The software Gradistat v.8^52^ was used to obtain the aforementioned parameters by the Folk & Ward method^53^. The method of moments for calculation of average, sorting, skewness and kurtosis was disregarded as it is enormously affected by outliers and should not be used unless the complete size distribution of sediment grains is fully known^54^.

Total nitrogen content (N) was determined using the Kjeldahl method as described by Page *et al*.^55^, and that of organic carbon (C) by the method of Anne^56^ adapted for a microplate reader. Finally, the C/N ratio was calculated. For a summary of granulometry, organic matter content and pH values per sample see Supplementary Information.

### Geometric morphometrics

**Figure 5:**
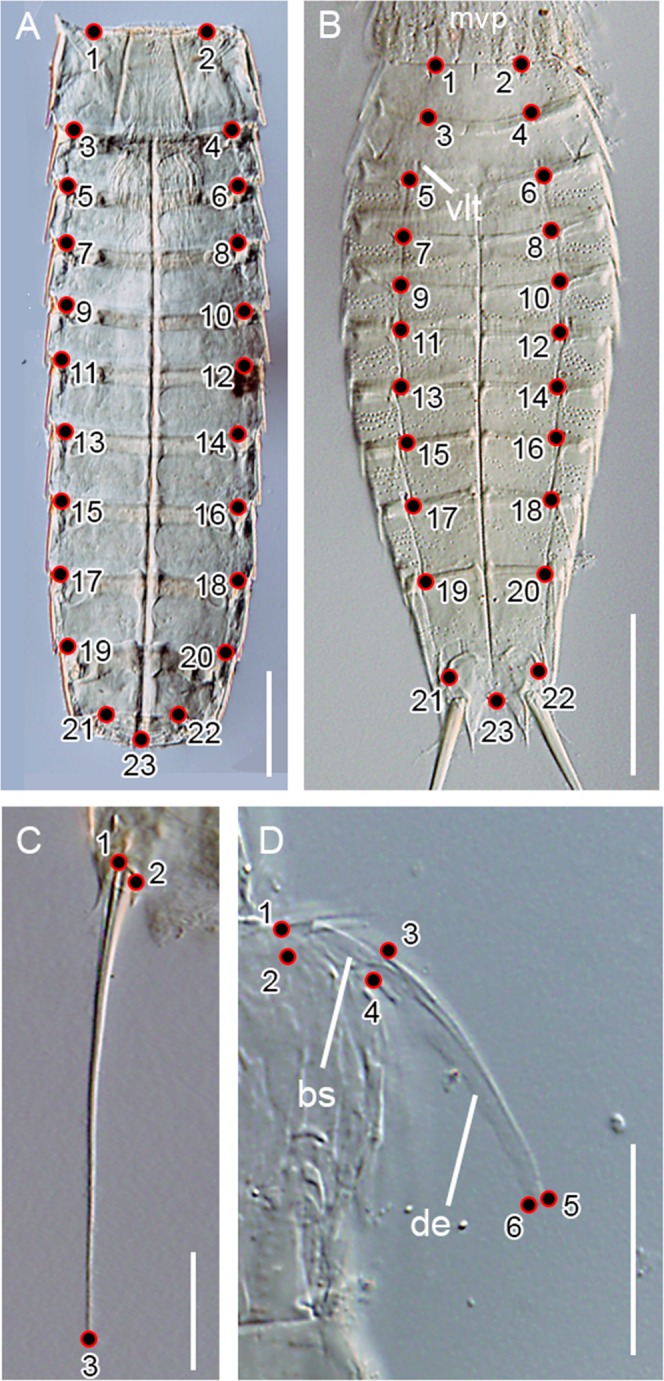
Position of landmarks used on digital micrographs. (**A**) Sternal plates of an adult male of *Pycnophyes communis* (Allomalorhagida); (**B**) sternal plates of an adult male of *Echinoderes* sp. 1 (Cyclorhagida); (**C**) right lateral terminal spine of an adult male of *Echinoderes* sp. 2 (Cyclorhagida); (**D**) primary spinoscalid of an adult male of *Echinoderes* sp. 1 (Cyclorhagida) in lateral view. Scales: A, 100 μm; B,C, 50 μm; D, 20 μm. Abbreviations: bs, basal sheath; de, distal end; mvp, midventral placid; vlt, ventrolateral tube.

Body, LTS and primary spinoscalids shape and size were analysed independently through geometric morphometrics. The software tpsUtil v.3.2 was used to build the tps files^57^. Placing of landmarks was done using the software tpsDig v.2.31^57^. For body shape, a total of 23 Cartesian landmarks were placed to extract the sternal plates morphology as reflection of body shape. When kinorhynchs are mounted for LM, the final shape of the body can be strongly biased by the force used to flatten the specimens in order to make the taxonomic characters more visible^25^. However, sternal plates’ morphology is not affected by this process, and hence they turn out as the best suitable feature to study general body shape in kinorhynchs. Landmarks 1–22 were positioned in each segment at the anterior margin of the pachycycli where tergal and sternal plates joint, whereas landmark 23 was placed at the posterior joint of the sternal plates of segment 11 (Fig. 5A,B). For species with a single, ring-like cuticular plate at the first trunk segment (*e.g*. genera *Echinoderes* and *Dracoderes*), landmarks were placed at the base of the two placids that are closest to the midventral placid (Fig. 5B). For species with a single, ring-like cuticular plate also at the second trunk segment (*e.g*. genus *Echinoderes*), landmarks were placed at the anterior margin of the pachycycli that is immediately above the lateroventral/ventrolateral tubes (Fig. 5B). For LTS’ shape, a total of three Cartesian landmarks were used to extract their morphology. Two landmarks were placed at the base of the spine, while the third one was positioned at the tip (Fig. 5C). For primary spinoscalids’ shape, six Cartesian landmarks were used: two at the base of the scalid, two at the junction between the basal sheath and the tip, and two at the tip of the scalid (Fig. 5D). Only primary spinoscalids laterally placed (Fig. 5D) were used in order to have all the photographs equally oriented.

MorphoJ v.1.07a software^58^ was used to superimpose the resulting landmark configuration by Generalized Procrustes analysis and to compute CS as the square root of the summed squared distances of each Cartesian landmark from the centroid of the landmark configuration. CS values are the only measurement independent of shape and represent the overall size of each studied structure^59^. For body shape, the Generalized Procrustes analysis was run taking into account the bilateral symmetry of kinorhynchs, defining a symmetric and an asymmetric component of the variation^58^. A prior series of multiple linear regression of Procrustes coordinates onto CS were used to test for allometry (*i.e*. influence of size on shape) as defined by Monteiro^60^. Influence of allometry was found in the symmetric component of body shape (*r*^2^ = 0.2501; *p* < 0.001) and in the LTS’ analysis (*r*^2^ = 0.4228; *p* < 0.001). Therefore, residuals of the regression were used instead of the raw coordinates in the subsequent analyses to correct for the allometric influence^61^. Principal component analyses (PCA) and wireframe graphs were performed to visualize the patterns of shape changes for each component.

### Statistical analyses

All the statistical analyses were implemented in R v.3.0.1. We firstly tested for correlation of sediment variables using the Kendall rank correlation coefficient^62^ with the *ggpubr* package^63^. Percentages of the three main categories of size particles were removed from the variables because of correlation: % of gravel correlated with average size (*tau* = 0.51; p = 0.01), % of sand with average size (*tau* = 0.47; p = 0.02), sorting (*tau* = −0.43; p = 0.03) and pH (*tau* = −0.40; p = 0.05), and % of mud with average size (*tau* = 0.57; p < 0.001), sorting (*tau* = 0.42; p = 0.03) and C/N (*tau* = 0.57; p = 0.03).

A series of linear mixed models (LMM) were used to assess the effect of the sediment structure and composition on body, LTS and primary spinoscalids shape. To control the effect of the phylogeny in our species dataset, we extract the phylogenetic information from Linnaean taxonomy using a nested structure in the random-effect component of the LMMs^64^. PC scores of the main PCA axes that define the variation in shape among individuals in geometric morphometrics were used as the response variables of the LMMs, whereas the sediment variables (average size, sorting, skewness and kurtosis) were set as the fixed-effect component. LMMs were performed using the *nlme* and *lme4* packages^65,66^. Marginal and conditional *R*^2^ were calculated for each LMM using the *MuMIn* package^67^.

When the assumptions of the LMMs were violated (*i.e*. absence of correlation in the residuals, homoscedasticity and normal distribution of the residuals), a series of generalized linear mixed models (GLMMs) were run. The Durbin-Watson, Breusch-Pagan and Shapiro-Wilk tests were used to check for correlation in the residuals, homoscedasticity and normal distribution of the residuals respectively, using the *lmtest* package^68^. GLMMs were run using the *glmmADMB* package^69^.

## Supplementary information

Supplementary Information.

## Data Availability

All data generated or analysed during this study are available from the corresponding author on reasonable request or included in this published article (and its Supplementary Information Files).
