# Supplementary material for: Does sediment composition sort kinorhynch communities? An ecomorphological approach through geometric morphometrics
Source: Sci Rep. 2020 Feb 13;10:2603. doi: 10.1038/s41598-020-59511-4 (PMC7018755; doi:10.1038/s41598-020-59511-4)
Supplement: Supplementary file 1 — Supplementary Information. [file 41598_2020_59511_MOESM1_ESM.pdf]

Does sediment composition sort kinorhynch communities? An ecomorphological approach through geometric morphometrics

Diego Cepeda[1,*], Dolores Trigo[1], Fernando Pardos[1,+], and Nuria Sánchez[2,+]

[1]Universidad Complutense, Department of Biodiversity, Ecology and Evolution, Madrid, 28040, Spain.

[2]Institut Français de Recherche pour l'Exploitation de la Mer, Deep-sea Laboratory, Plouzané, 29280, France.

[*]diegocepeda@ucm.es

[+]these authors contributed equally to this work

Supplementary Material

Supplementary Material 1. Information on sampling stations and species and number of specimens per species found in each locality that were used in the present study. Abbreviations: *n*, number of specimens per species.

Locality	Date	Coordinates	Depth	Species	*n*
Algeciras Bay 1	11.02.07	36º05.805'N 5º26.284'W	30	*Echinoderes cantabricus*	1
				Echinoderes hispanicus	4
				Echinoderes sp. 1	6
Algeciras Bay 2	11.02.07	36º07.229'N 5º25.114'W	24	*Echinoderes sp. 1*	3
Algeciras Bay 3	11.02.08	36º09.272'N 5º26.296'W	12	*Echinoderes hispanicus*	1
				Setaphyes dentatus	8
Algeciras Bay 4	11.02.08	36º10.348'N 5º26.464'W	16	*Dracoderes gallaicus*	3
Algeciras Bay 5	11.02.08	36º10.583'N 5º24.620'W	25	*Echinoderes cantabricus*	2
Algeciras Bay 6	11.02.08	36º10.741'N 5º23.243'W	8	*Echinoderes dujardinii*	1
				Echinoderes hispanicus	1
				Setaphyes dentatus	3
Algeciras Bay 7	11.02.08	36º09.630'N 5º22.256'W	12	*Echinoderes dujardinii*	2
				Echinoderes hispanicus	6
Isla Cristina 1	11.04.11	37º11.940'N 7º21.236'W	2	*Echinoderes dujardinii*	2
				Setaphyes dentatus	1
Isla Cristina 2	11.04.11	37º10.963'N 7º16.549'W	11	*Pycnophyes communis*	2
				Setaphyes dentatus	6
Isla Cristina 3	11.04.11	37º11.527'N 7º14.601'W	12	*Setaphyes dentatus*	5
Isla Cristina 4	11.04.11	37º12.320'N 7º20.534'W	4	*Setaphyes dentatus*	5
Isla Cristina 5	11.04.11	37º11.887'N 7º13.019'W	7	*Setaphyes dentatus*	6
Isla Cristina 6	11.04.12	37º08.324'N 7º20.308'W	15	*Pycnophyes communis*	2
Cádiz 1	11.11.10	36º33.755'N 6º18.500'W	13	*Leiocanthus lageria*	1
				Pycnophys communis	7
				Setaphyes dentatus	2
Cádiz 2	11.11.10	36º35.791'N 6º17.888'W	10	*Setaphyes dentatus*	3

Cádiz 3	11.11.10	36º34.117’N	7	*Echinoderes dujardinii*	1
		6º15.141’W		*Pycnophyes communis*	1
				Setaphyes dentatus	1
Cádiz 4	11.11.10	36º32.761’N	11	*Echinoderes hispanicus*	2
		6º15.141’W		*Pycnophyes almansae*	1
				Pycnophyes communis	3
				Setaphyes dentatus	1
Cádiz 5	11.11.10	36º32.310’N	4	*Echinoderes worthingi*	1
		6º14.245’W			
Cádiz 6	11.11.10	36º31.930’N	4	*Echinoderes dujardinii*	2
		6º12.960ºW		*Echinoderes worthingi*	1
Cádiz 7	11.11.11	36º31.124’N	11	*Echinoderes dujardinii*	2
		6º15.692’W		*Echinoderes hispanicus*	1
				Echinoderes sp. 1	1
Cádiz 8	11.11.11	36º28.304’N	4	*Echinoderes hispanicus*	1
		6º10.936’W			
Cádiz 9	11.11.11	36º29.798’N	1	*Echinoderes cantabricus*	4
		6º12.871’W		*Echinoderes hispanicus*	1
				Leiocanthus lageria	1
Kingston	76.03.10	17º56.24’N	2	*Echinoderes imperforatus*	1
Harbor 1		76º50.00’W		*Echinoderes parahorni*	1
				Echinoderes sp. 2	2
Kingston	76.03.10	17º56.30’N	1	*Echinoderes parahorni*	3
Harbor 2		76º49.12’W		*Echinoderes sp. 2*	3
Kingston	76.03.11	17º57.18’N	4	*Echinoderes astridae*	6
Harbor 3		76º50.24’W		*Echinoderes parahorni*	3

Supplementary Material 2

Table 1. Granulometry of sediment samples. Abbreviations: *K*, kurtosis; *Sk*, skewness; *X*, average size; *σ*, sorting.

**Sample**	**Textural group**	**%Gravel**	**%Sand**	**%Mud**	**X**	***σ***	***Sk***	***K***
Algeciras Bay 1	Slightly gravelly sand	0.6	91.,6	7.8	229.5	3.488	-0.262	2.103
Algeciras Bay 2	Muddy sandy gravel	66.8	23.5	9.8	1353.9	3.699	-1.644	4.463
Algeciras Bay 3	Slightly gravelly sand	0	93.1	6.9	133.7	3.007	-0.301	2.536
Algeciras Bay 4	Sligthly gravelly sandy mud	0	31.2	68.8	10.42	7.011	0.286	0.557
Algeciras Bay 5	Sligthly gravelly sandy mud	0	49.9	50.1	23.56	7.072	-0.566	0.519
Algeciras Bay 6	Sligthly gravelly sand	0.1	94.9	5	210.5	1.918	-0.273	0.869
Algeciras Bay 7	Gravelly sand	9.5	82.5	7.9	292.3	4.313	0.024	2.254
Cádiz 1	Sligthly gravelly muddy sand	0	88.6	11.4	126.3	1.712	-0.325	0.923
Cádiz 2	Sligthly gravelly muddy sand	0	82.7	17.3	99.54	2.725	-0.269	2.679
Cádiz 3	Sligthly gravelly muddy sand	0	82.6	17.4	101.9	2.735	-0.268	2.674
Cádiz 4	Sligthly gravelly sand	0	95.8	4.2	199.4	1.863	-0.081	0.954
Cádiz 5	Sligthly gravelly sand	0	92.8	7.1	140.9	2.531	-0.373	2.22
Cádiz 6	Gravelly sand	7.2	88.6	4.1	327.8	2.638	0.038	2.368
Cádiz 7	Sligthly gravelly sand	2.4	90.5	7.1	305.2	2.798	-0.415	3.384
Cádiz 8	Sligthly gravelly sandy mud	0	41.8	58.1	16.06	8.372	0.181	0.537
Cádiz 9	Sligthly gravelly mud	3.2	4.7	92.1	19.85	3.146	-0.389	2.152
Isla Cristina 1	Muddy gravel	38.9	11.1	50	56.96	16.54	-0.088	0.594

Isla Cristina 2	Sligthly gravelly muddy sand	3.2	80.9	15.9	108.6	2.658	-0.164	2.301
Isla Cristina 3	Sligthly gravelly muddy sand	0	86.7	13.3	114.3	1.677	-0.005	0.778
Isla Cristina 4	Sligthly gravelly sand	0	93.5	6.5	163.9	2.238	-0.15	1.103
Isla Cristina 5	Sligthly gravelly muddy sand	0	86.6	13.4	122.7	1.901	0.138	0.913
Isla Cristina 6	Gravelly mud	7.2	15.7	77.1	19.52	7.639	-0.036	1.541
Kingston Harbor 1	Sligthly gravelly sandy mud	0	11	89	9.502	4.639	-0.225	0.609
Kingston Harbor 2	Sligthly gravelly mud	0	2.8	97.2	27.52	2.09	-0.437	2.367
Kingston Harbor 3	Sligthly gravelly muddy sand	0	59.9	40	49.44	3.715	-0.468	1.198

Table 2. Organic matter content and pH of sediment samples. Abbreviations: *C*, carbon; C/N, carbon-nitrogen ratio, *N*, nitrogen; *NC*, not calculated.

**Sample**	***%C***	***%N***	**C/N**	**pH**
Algeciras Bay 1	0.8624	0.0423	20.39	8.311
Algeciras Bay 2	1.0984	0.0849	12.94	8.883
Algeciras Bay 3	0.3722	0.0204	18.25	8.084
Algeciras Bay 4	2.2821	0.1821	12.53	6.691
Algeciras Bay 5	2.2356	0.1649	13.56	6.677
Algeciras Bay 6	0.3397	0.0141	24.09	8.311
Algeciras Bay 7	0.6805	0.0646	10.53	8.883
Cádiz 1	0.4309	0.0359	12	8.349
Cádiz 2	0.4633	0.0193	24.01	8.432
Cádiz 3	0.7522	0.0246	30.58	8.027
Cádiz 4	0.5392	0.0157	34.34	8.293
Cádiz 5	0.5452	0.0089	61.26	8.403
Cádiz 6	0.8622	0.0171	50.42	8.219
Cádiz 7	0.6309	0.0377	18.72	8.252
Cádiz 8	1.8445	0.0789	23.38	7.419
Cádiz 9	*NC*	*NC*	*NC*	7.681
Isla Cristina 1	1.2274	0.0758	16.19	7.543
Isla Cristina 2	0.6058	0.0312	19.42	7.942

Isla Cristina 3	0.3232	0.0158	20.46	7.775
Isla Cristina 4	0.4708	0.0195	24.14	8.081
Isla Cristina 5	0.3717	0.0234	15.88	8.3
Isla Cristina 6	1.5441	0.1415	10.91	6.84
Kingston Harbor 1	*NC*	*NC*	*NC*	7.53
Kingston Harbor 2	5.3474	0.6932	7.71	*NC*
Kingston Harbor 3	4.3655	0.2906	15.02	7.877
